# Diagnostic accuracy of the Clock Drawing Test in screening for early post-stroke neurocognitive disorder: the Nor-COAST study

**DOI:** 10.1186/s12883-023-03523-w

**Published:** 2024-01-09

**Authors:** Egle Navickaite, Ingvild Saltvedt, Stian Lydersen, Ragnhild Munthe-Kaas, Hege Ihle-Hansen, Ramune Grambaite, Stina Aam

**Affiliations:** 1 Department of Neuromedicine and Movement Science, Norwegian University of Science and Technology, Faculty of Medicine and Health Science, Trondheim, Norway; 2 Department of Geriatric Medicine, Clinic of Medicine, St. Olavs Hospital, Trondheim University Hospital, Trondheim, Norway; 3 Department of Mental Health, Norwegian University of Science and Technology, Faculty of Medicine and Health Science, Trondheim, Norway; 4 Department of Medicine, Kongsberg Hospital, Vestre Viken Hospital Trust, Kongsberg, Norway; 5 Department of Medicine, Bærum Hospital, Vestre Viken Hospital Trust, Drammen, Norway; 6 Stroke Unit, Department of Neurology, Oslo University Hospital, Ullevaal, Oslo, Norway; 7 Department of Medical Research, Bærum Hospital, Vestre Viken Hospital Trust, Drammen, Norway; 8 Department of Psychology, Norwegian University of Science and Technology, Trondheim, Norway

**Keywords:** Post-stroke neurocognitive disorder, Post-stroke cognitive impairment, Clock Drawing Test, Screening

## Abstract

**Background:**

Post-stroke neurocognitive disorder, though common, is often overlooked by clinicians. Moreover, although the Montreal Cognitive Assessment (MoCA) has proven to be a valid screening test for neurocognitive disorder, even more time saving tests would be preferred. In our study, we aimed to determine the diagnostic accuracy of the Clock Drawing Test (CDT) for post-stroke neurocognitive disorder and the association between the CDT and MoCA.

**Methods:**

This study is part of the Norwegian Cognitive Impairment After Stroke study, a multicentre prospective cohort study following patients admitted with acute stroke. At the three-month follow-up, patients were classified with normal cognition, mild neurocognitive disorder, or major neurocognitive disorder according to the Diagnostic and Statistical Manual of Mental Disorders, Fifth Edition criteria. Any neurocognitive disorder compromised both mild- and major neurocognitive disorder. The CDT at the three-month assessment was given scores ranging from 0 to 5. Patients able to complete the CDT and whose cognitive status could be classified were included in analyses. The CDT diagnostic accuracy for post-stroke neurocognitive disorder was identified using receiver operating characteristic curves, sensitivity, specificity, positive predictive value, and negative predictive value. The association between the MoCA and CDT was analysed with Spearman’s rho.

**Results:**

Of 554 participants, 238 (43.0%) were women. Mean (SD) age was 71.5 (11.8) years, while mean (SD) National Institutes of Health Stroke Scale score was 2.6 (3.7). The area under the receiver operating characteristic curve of the CDT for major neurocognitive disorder and any neurocognitive disorder was 0.73 (95% CI, 0.68–0.79) and 0.68 (95% CI, 0.63–0.72), respectively. A CDT cutoff of < 5 yielded 68% sensitivity and 60% specificity for any neurocognitive disorder and 78% sensitivity and 53% specificity for major neurocognitive disorder. Spearman’s correlation coefficient between scores on the MoCA and CDT was 0.50 (95% CI, 0.44–0.57, *p* < .001).

**Conclusions:**

The CDT is not accurate enough to diagnose post-stroke neurocognitive disorder but shows acceptable accuracy in identifying major neurocognitive disorder. Performance on the CDT was associated with performance on MoCA; however, the CDT is inferior to MoCA in identifying post-stroke neurocognitive disorder.

**Trial registration:**

ClinicalTrials.gov (NCT02650531). Retrospectively registered January 8, 2016.

**Supplementary Information:**

The online version contains supplementary material available at 10.1186/s12883-023-03523-w.

## Background

Although the incidence of stroke has decreased, increased longevity, population ageing, and the improved survival of stroke patients have increased the prevalence of stroke survivors [1]. Half of all survivors develop cognitive impairment following stroke [2], which increases the burden for them, their relatives and the healthcare system [3–5].

The Diagnostic and Statistical Manual of Mental Disorders, Fifth Edition (DSM-5) [6] has introduced the terms mild- and major neurocognitive disorder (NCD), replacing the traditionally used terms mild cognitive impairment and dementia, respectively. Clinical experience has shown that post-stroke NCD is often overlooked in stroke care, often due to the lack of time and capacity needed to perform extensive evaluation of all stroke survivors. In response, a brief and valid screening tool could be the first step to identify patients who need more comprehensive diagnostic assessment and tailored follow-up [7].

In assessing post-stroke NCD, the Mini Mental State Exam (MMSE) exhibits a ceiling effect, and its diagnostic accuracy among stroke survivors is debated [8–10]. The Montreal Cognitive Assessment (MoCA), by contrast, has shown more promising results [8, 11–13]. Although the MoCA was developed as a 10-min screening tool [14], clinicians experience that it often is more lengthy to perform. To minimise the risk of overlooking post-stroke NCD in busy clinical practice, less time-consuming screening tools would be favourable, such as the Clock Drawing Test (CDT) [15]. The CDT assesses a variety of cognitive domains, including perceptual-motor function, executive function, complex attention (selective and sustained), memory (semantic), and language (receptive) [16–18]. Its diagnostic accuracy for non-stroke NCD is well documented with good intra- and interrater reliability [19, 20]. To our knowledge, only one previous study has examined the diagnostic accuracy of the CDT for post-stroke NCD, presenting adequate levels of sensitivity and specificity [21]. The study’s sample of 98 participants was small. Therefore, a replication of this study with a larger sample size is requested.

We aimed to determine the diagnostic accuracy of the CDT as a screening tool for post-stroke NCD diagnosed according to the DSM-5 criteria, and the association between patients’ CDT score and MoCA score three months post-stroke.

## Methods

Our study is part of the Norwegian Cognitive Impairment After Stroke (Nor-COAST) study, a multicentre prospective cohort study involving 815 patients admitted with acute stroke at five hospitals in Norway [22]. Consecutive patients with confirmed diagnoses of acute stroke, were included during the initial hospital stay. The inclusion criteria were admission with acute stroke within seven days of symptom debut to a stroke unit in one of the participating hospitals, being at least 18 years old and being able to communicate in a Scandinavian language. Exclusion criteria were a life expectancy less than three months, as clinically assessed by trained study nurses and stroke physicians. Participant recruitment proceeded from May 2015 through May 2017, and participants were followed up at three, 18 and 36 months after the stroke. The Nor-COAST study is described in greater detail elsewhere [22, 23]. Patients able to complete the CDT as part of the MoCA and whose cognitive status could be classified were included in our study.

### Baseline characteristics

Information about the patients’ socio-demographic characteristics, medical history and premorbid function was collected during their initial hospital stays from medical records and through interviews with participants and/or caregivers. The modified Rankin Scale, with scores 0–6, was used to evaluate global function before and after stroke [24, 25], while activities of daily living were assessed using the Barthel Index [26]. Stroke severity was evaluated using the National Institutes of Health Stroke Scale (NIHSS) [27], on the day after admission, whereas ischemic stroke subtype was classified according to the Trial of ORG 10172 in Acute Stroke Treatment (TOAST) classification [28]. Last, information about pre-stroke cognitive impairment was assessed by trained nurses using the Global Deterioration Scale [29], originally developed for assessment of primary degenerative dementia, but proven valid also for vascular dementia [30, 31].

### Cognitive assessments

At the three-month follow up, participating patients underwent cognitive assessment with a neurocognitive test battery and the MoCA at the hospitals’ outpatient clinics. The neurocognitive test battery was based on recommendations from the National Institute of Neurological Disorders and Stroke-Canadian Stroke Network [32], the 30-min version. The tests used had previously been translated into Norwegian and assessed the following cognitive domains: complex attention (Trail Making Test A [33]), executive function (Trail Making Test B [33] and Verbal Fluency test letter [FAS] [34, 35]), memory (Word List Recall [36]), language (Verbal Fluency Test Category-Animals [37]), and perceptual-motor function (visuospatial/executive subtest of MoCA [14]). Social cognition was not assessed. In previous work in the Nor-COAST study, cognitive status was classified according to the DSM-5 criteria for NCD [23]. NCD was defined as a score less than − 1.5 standard deviation (SD) in at least one cognitive domain. Mild NCD was defined as NCD and independence in instrumental activities of daily living and major NCD as NCD and dependency in instrumental activities of daily living [23].

All participants were assessed by the MoCA, a global cognitive screening test with maximum score of 30, with an additional point for education ≤ 12 years [14]. After initial testing, the CDT tasks from MoCA were rescored in accordance with a 6-point Norwegian scoring method, previously published by Strobel et al., and commonly used by Norwegian clinicians ([38, 39], for English adaptation please see Supplementary Material, Table S1 and Fig. S1-S4). In Strobel et al.’s scoring method, participants are handed a pre-printed circle used to draw a clock. In the CDT task of MoCA, participants are instructed to draw the circle by hand. In accordance with the scoring method by Strobel et al., the ability to draw a circle was not assessed. Strobel et al. recommend a cutoff < 4 for the diagnosis of NCD. To investigate the impact of different cutoff values on the CDT’s diagnostic accuracy for post-stroke NCD, we performed analyses with two cutoff values: < 4 (i.e., scores of 0–3 indicating NCD, and scores of 4–5 indicating normal cognition) and < 5 (i.e., scores 0–4 indicating NCD, and a score of 5 indicating normal cognition).

### Statistics

To minimise bias and the loss of sample size due to excluded participants, missing items in the MoCA scores were imputed by the mean of the available MoCA items for the same participant. The three-category cognitive status was dichotomised into normal cognition and any NCD (i.e., mild- or major NCD) and into normal cognition/mild NCD and major NCD [40]. The CDT’s accuracy in diagnosing any NCD and major NCD was quantified in terms of sensitivity and specificity for the CDT score cutoff values of < 4 and < 5, as well as in terms of positive predictive value and negative predictive value. Confidence intervals (CI) for proportions were calculated using the Wilson score method [41]. We also calculated the area under the receiver operating characteristic curve (AUC) for both any- and major NCD. We interpret AUC values as suggested by Hosmer et al. [42] as 0.5 to 0.7 indicating poor discrimination, 0.7 to 0.8 as acceptable, 0.8 to 0.9 as excellent, and above 0.9 as outstanding. The association between scores on the MoCA and CDT was quantified using Spearman’s correlation. All statistical analyses were performed using IBM SPSS Statistics version 28.0.1.

## Results

**Fig. 1:**
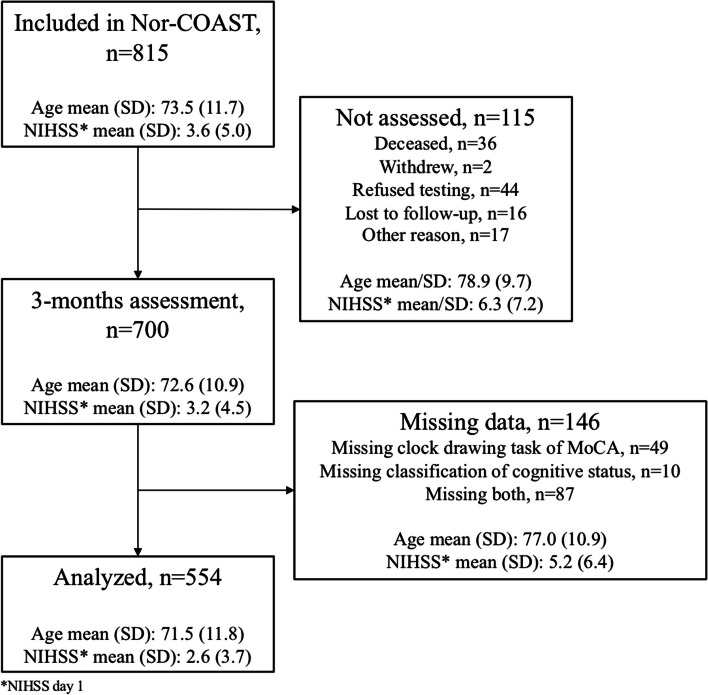
Flowchart for inclusion of participants *Nor-COAST* Norwegian Cognitive Impairment After Stroke study, *NIHSS* National Institutes of Health Stroke Scale, *MoCA*, Montreal Cognitive Assessment, *SD* standard deviation

As shown by Kuvås et al. [43], 2505 patients with the diagnosis of stroke were admitted to the participating hospitals during the recruitment period, of these, 559 were not eligible. The rest were not included because they were declining participation, discharged early, failed to be screened or for other reasons. Of the 815 included participants in the Nor-COAST study, 261 were not assessed or had missing data at three-months. These tended to be older and to have more severe strokes (Fig. 1).

Our study included 554 participants able to complete the CDT task from the MoCA and whose cognitive status could be classified at the three-month follow-up. All included participants were able to draw a circle for the clock. Of them, 238 (43.0%) were women, the mean (SD) age was 71.5 (11.8) years, and patients suffered mostly minor strokes (Table 1). Pre-stroke Global Detoriation Scale scores revealed that few patients had NCD prior to stroke. At three months post-stroke, 253 participants (45.7%) had normal cognition, 161 (29.1%) had mild NCD and 140 (25.3%) had major NCD according to the DSM-5 criteria [23].

**Table 1:** Participants’ characteristics at baseline and cognitive status at three months

**Demographics**	*n* = 554	
Age in years, mean (SD)		71.5 (11.8)
Female sex, n (%)		238 (43.0)
Education in years, mean (SD)		12.5 (3.8)
**Stroke subtype, n (%)**		
Infarction		504 (91.0)
Haemorrhage		50 (9.0)
**TOAST classification, n (%)**	*n* = 488/504	
Large-vessel disease		49 (8.8)
Cardioembolic disease		112 (20.2)
Small-vessel disease		113 (20.4)
Other aetiology		13 (2.3)
Undetermined aetiology		201 (36.3)
**Treatment, n (%)**		
Thrombolysis		129 (23.3)
Thrombectomy		11 (2.0)
**Function, mean (SD)**		
NIHSS score, day 1 (0–42)	*n* = 542	2.6 (3.7)
Pre-stroke mRS score (0–6)	*n* = 552	0.8 (1.0)
mRS score at discharge (0–6)	*n* = 552	2.0 (1.3)
Barthel Index (0–100)	*n* = 553	89.2 (18.0)
Pre-stroke GDS score (0–7), mean (SD) 3, mild NCD, n (%) 4–7, major NCD, n (%)	*n* = 549	1.4 (0.8) 33 (6.0) 19 (3.5)
**Cognitive status at three months, n (%)**		
Normal cognition		253 (45.7)
Mild NCD		161 (29.1)
Major NCD		140 (25.3)
**CDT score, n (%)**		
0–3		139 (25.1)
4		165 (29.8)
5		250 (45.1)

*TOAST* Trial of ORG 10172 in Acute Stroke Treatment, *NIHSS* National Institutes of Health Stroke Scale, *mRS* modified Rankin Scale, *GDS* Global Deterioration Scale, *NCD* neurocognitive disorder, *CDT* Clock Drawing Test, *SD* standard deviation

### Diagnostic accuracy of the Clock Drawing Test (CDT)

Cross-tabulations for the CDT score cutoff values of < 4 and < 5 and the three-category cognitive status are shown in Table 2. Using CDT cutoff < 4, 129 and 64 participants with any- and major NCD, respectively, were false negatives due to having CDT scores above the cutoff value. A CDT cutoff < 5 identified a larger number of participants with NCD; however, the number of false positives increased to 100. The corresponding measures for diagnostic accuracy are shown in Table 3. Sensitivity for major NCD given a cutoff value of < 5 was 78% (95% CI, 70–84), whereas specificity was 53% (95% CI, 48–58). For any NCD, a CDT cutoff < 5 yielded a sensitivity of 68% (95% CI, 62–73) and a specificity of 61% (95% CI, 54–66). As expected, a CDT cutoff < 4 yielded lower sensitivity but higher specificity for both any- and major NCD (Table 3).

**Table 2:** Comparison of Clock Drawing Test (CDT) and cognitive status classified according to the DSM-5 criteria

CDT cut-off score	Normal cognition	Mild NCD	Major NCD	Total, n (%)
< 4, n (%)	31 (22.3)	32 (23.0)	76 (54.7)	139 (100)
≥ 4, n (%)	222 (53.5)	129 (31.1)	64 (15.4)	415 (100)
Total, n (%)	253 (45.7)	161 (29.1)	140 (25.3)	554 (100)
< 5, n (%)	100 (32.9)	95 (31.3)	109 (25.9)	304 (100)
5, n (%)	153 (61.2)	66 (26.4)	31 (12.4)	250 (100)
Total, n (%)	253 (45.7)	161 (29.1)	140 (25.3)	554 (100)

*DSM-5* Diagnostic and Statistical Manual of Mental Disorders, Fifth Edition, *NCD* neurocognitive disorder

*n* = 554

**Table 3:** Diagnostic accuracy of the Clock Drawing Test (CDT) for the diagnosis of neurocognitive disorder (NCD)

CDT cut-off score	Sensitivity (%)	95% CI	Specificity (%)	95% CI	PPV (%)	95% CI	NPV (%)	95% CI
Any NCD								
< 4	36	31–41	88	83–91	78	70–84	54	49–58
< 5	68	62–73	61	54–66	67	62–72	61	55–67
Major NCD								
< 4	54	46–62	85	81–88	55	46–63	85	81–88
< 5	78	70–84	53	48–58	36	31–41	88	83–91

*PPV* positive predictive value, *NPV* negative predictive value, *CI* confidence interval

*n* = 554

**Fig. 2:**
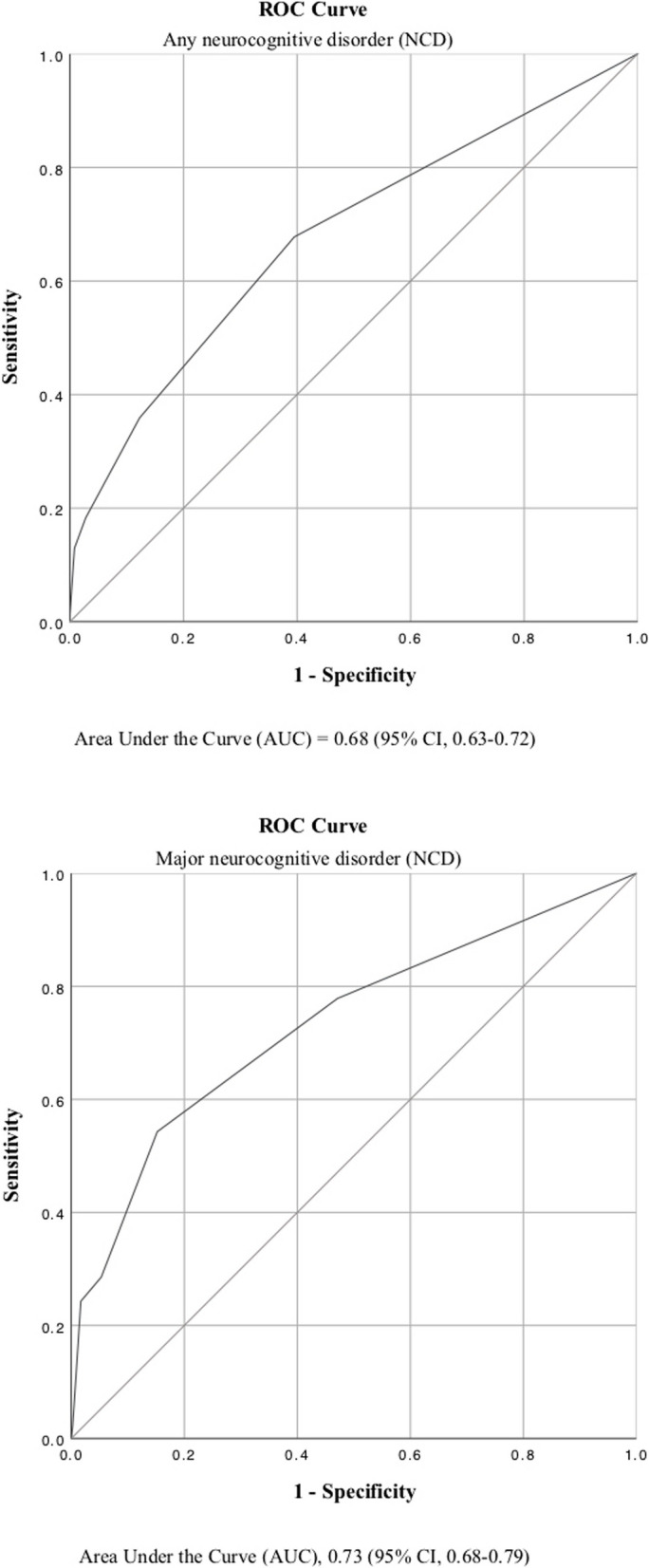
Receiver operating characteristic (ROC) curves of the Clock Drawing Test for neurocognitive disorder

Receiver operating characteristic (ROC) curves of the CDT for any- and major post-stroke NCD with corresponding AUC values are shown in Fig. 2.

### Association between the Montreal Cognitive Assessment (MoCA) and Clock Drawing Test (CDT)

Spearman’s correlation coefficient between MoCA and CDT scores was 0.50 (95% CI, 0.44–0.57). Table 4 shows the mean MoCA scores for patients with CDT scores 0–5 and CDT scores above or below the cutoff values of < 4 and < 5, respectively. Patients receiving lower scores on the CDT tend to receive lower scores on the MoCA.

**Table 4:** Montreal Cognitive Assessment (MoCA) scores versus Clock Drawing Test (CDT) scores

CDT score	Mean (SD) MoCA score	n
0	11.3 (6.2)	4
1	15.2 (5.6)	35
2	21.7 (5.4)	21
3	21.9 (4.3)	77
4	24.8 (3.2)	165
5	26.3 (2.7)	250
CDT cut-off score	Mean (SD) MoCA score	n
< 4	19.8 (5.8)	137
≥ 4	25.7 (3.0)	415
< 5	22.5 (5.2)	302
5	26.3 (2.7)	250

*SD* standard deviation

## Discussion

For the purpose of identifying patients with post-stroke NCD, the CDT demonstrated acceptable diagnostic accuracy for major NCD but failed to identify a substantial number of patients with any NCD (i.e., mild and major NCD). Moreover, despite an observed association between performance on the CDT and performance on the MoCA, the CDT is inferior to MoCA in screening for post-stroke NCD.

The purpose of a cognitive screening test is to identify patients who need a more comprehensive assessment for NCD, with sensitivity being of greater importance than specificity. In our study, the best balance between sensitivity and specificity was achieved by employing a CDT cutoff < 5. The CDT proved to have acceptable diagnostic accuracy for major NCD; however, its sensitivity for any NCD was lower with a similarly lower AUC. Thus, in using only the CDT, healthcare professionals are liable to overlook a substantial proportion of patients with mild NCD. If undetected, mild NCD is associated with an increased risk of reduced quality of life and poorer prognosis due to weak adherence to medication and a lack of tailored cognitive and physical stimulation that could prevent further cognitive decline [5, 44–46].

To our knowledge, only one study to date has investigated the CDT’s accuracy in diagnosing NCD among stroke survivors. In that research, Cova et al. observed a sensitivity of 80%, specificity of 76% and an AUC of 0.86 [21]. Several methodological differences between our studies might explain the divergence in our results. For one, Cova et al. employed a rather more comprehensive scoring system for the CDT, one with scores ranging from 0 to 13. For another, patients were evaluated in the acute phase following stroke, and the diagnosis of NCD was based on clinical judgment of the evaluating neurologist, not on the results of a neurocognitive test battery. As part of the Nor-COAST study, Munthe-Kaas studied the diagnostic accuracy of MoCA for NCD [12]. In that research, the MoCA had an AUC approaching 0.80 with 71% sensitivity and 73% specificity for post-stroke NCD, and other studies have revealed similar results [13, 21, 47, 48]. Compared to the results of our study, the CDT is inferior to the MoCA as a screening tool for post-stroke NCD.

All cognitive domains mentioned in DSM-5 except social cognition were assessed in the Nor-COAST study. Of these, all, except motor function were impaired, with memory most severely affected [2]. Although the CDT requires semantic memory regarding what a clock look like, it is better at assessing visuospatial and executive impairments [16–18]. This might somewhat explain why so many patients with NCD earn CDT scores above the cutoff values (i.e., false negatives). Ideally, to achieve high sensitivity for post-stroke NCD, a cognitive screening tool should assess all six cognitive domains [49]. In that light, the CDT falls short of the MoCA, which provides a more extensive cognitive evaluation [14, 50]. However, the CDT might be less influenced than the MoCA by verbal impairments, which are highly prevalent after stroke [51]. For that reason, the CDT may be more feasible among stroke patients with impairments in verbal expression following stroke.

A major strength of our study was its multicentre design with a high number of participants from five stroke units in different health regions in Norway [43]. Another strength was that we defined NCD according to the DSM-5 criteria and assessed patients using a neurocognitive test battery recommended for stroke patients [23, 32]. Finally, the CDT is generally quick and easy to administer and widely used in clinical practice, and we applied a scoring system well-known to many clinicians.

Our findings have some limitations. Although, the Nor-COAST population can be regarded as representative of the majority of the stroke population [43], patients included in the present study were younger and had milder strokes, which lowers their risk for post-stroke NCD. This may have affected the applicability of our results to the general stroke population. As expected, CDT and MoCA scores were associated, but we compared the MoCA with a sub-test of MoCA, which might have weakened the credibility of our results. However, we applied a different scoring method, which may have reduced the significance of this. An optimal study design would be to perform the entire CDT in accordance with Strobel’s method, including giving participants a pre-drawn circle and the ability to redo the test if unsatisfied. In this study, participants were instructed to draw a circle. These were sometimes small or uneven, and the numbers and hands of the clock were sometimes poorly aligned, which in our scoring method meant lower scores [38]. This could have increased the rate of false positives.

## Conclusion

Our findings suggest that although the CDT has acceptable diagnostic accuracy for major NCD following stroke, many patients with mild impairments are likely to be overlooked. The CDT is inferior to the MoCA in screening for post-stroke NCD, and though the CDT is better than no screening whatsoever, based on the present study, we do not recommend it as a routine screening test for post-stroke NCD. Clinicians should rely on other neurocognitive tests, such as MoCA. It may be hypothesized that the CDT applied together with reports on subjective cognitive difficulties or a brief neurocognitive test assessing memory, would increase diagnostic accuracy for post-stroke NCD. Further research on these subjects is needed, as well as on which specific domains are associated with impaired CDT scores.

### Supplementary Information

**Additional file 1.** Scoring table for CDT, English adaptation 2023 and Clock Drawing Test Visual Scoring Templates, English adaptation 2023.

## Data Availability

The datasets used in the study are not publicly available but are available from the corresponding author upon reasonable request.

## Abbreviations

Nor-COAST study: Norwegian Cognitive Impairment After Stroke study
NCD: Neurocognitive disorder
CDT: Clock Drawing Test
MoCA: Montreal Cognitive Assessment
MMSE: Mini Mental State Examination
DSM-5: Diagnostic and Statistical Manual of Mental Disorders, Fifth Edition
NIHSS: National Institutes of Health Stroke Scale
ROC curve: Receiver operating characteristic curve
AUC: Area under the receiver operating characteristic curve
CI: Confidence interval
SD: Standard deviation
