# Supplementary material for: Diagnostic accuracy of the Clock Drawing Test in screening for early post-stroke neurocognitive disorder: the Nor-COAST study
Source: BMC Neurol. 2024 Jan 9;24:22. doi: 10.1186/s12883-023-03523-w (PMC10775614; doi:10.1186/s12883-023-03523-w)
Supplement: Supplementary file 1 — Additional file 1. Scoring table for CDT, English adaptation 2023 and Clock Drawing Test Visual Scoring Templates, English adaptation 2023. [file 12883_2023_3523_MOESM1_ESM.docx]

SUPPLEMENTARY MATERIAL

Diagnostic Accuracy of the Clock Drawing Test in Screening for Early Post-Stroke Neurocognitive Disorder: The Nor-COAST Study

**Table** S1 Scoring table for CDT, English adaptation 2023 (1)

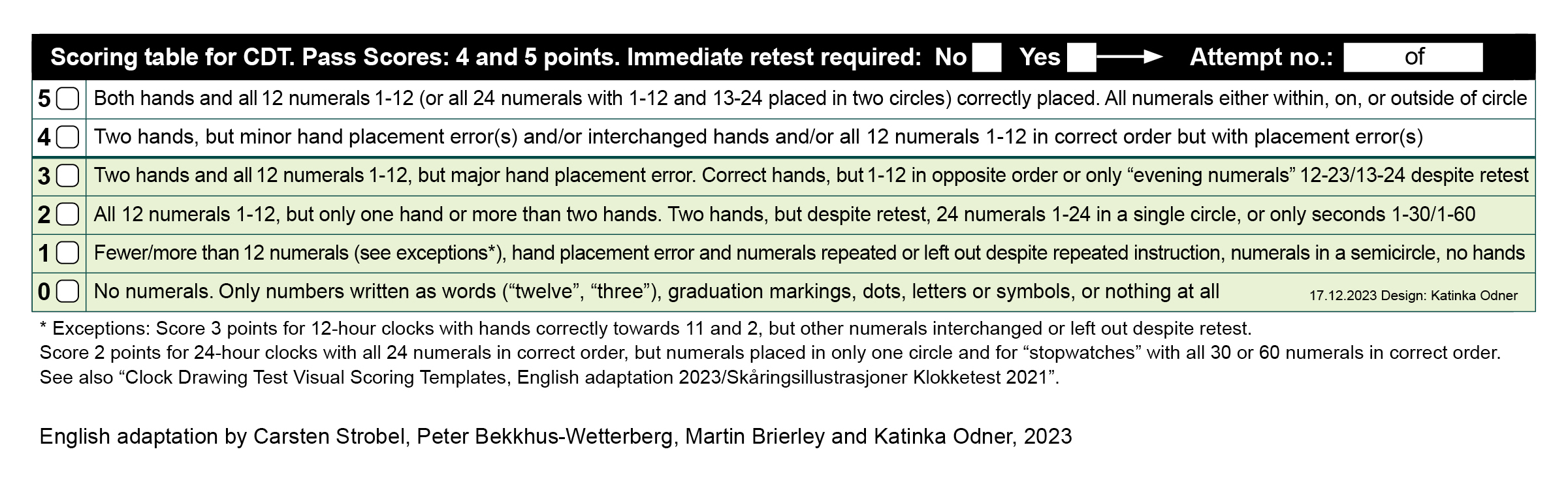


English adaptation by Carsten Strobel, Peter Bekkhus-Wetterberg, Martin Brierly and Katinka Odner.

**Figure S1-S4** Clock Drawing Test Visual Scoring Templates, English adaptation 2023 (2)

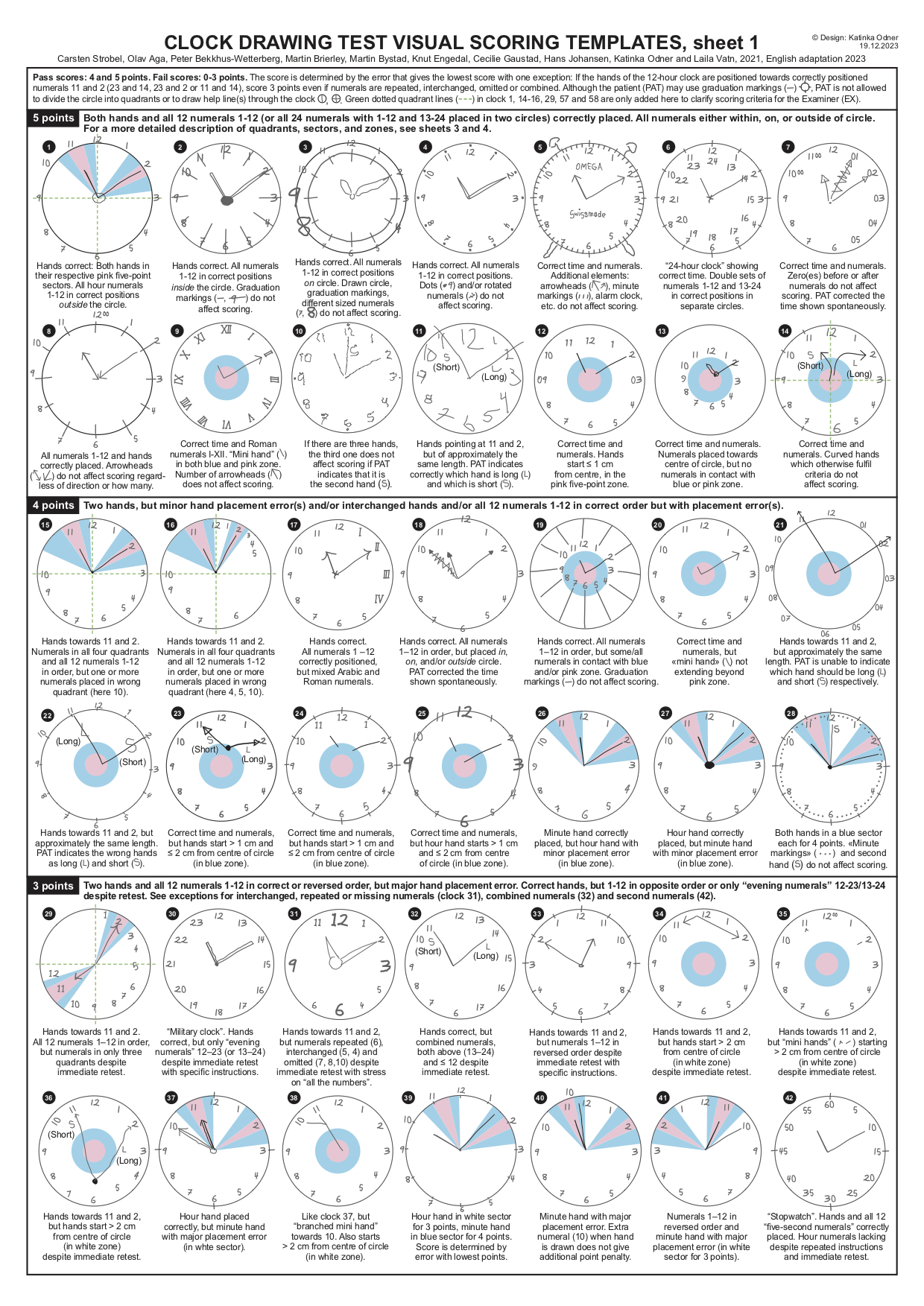


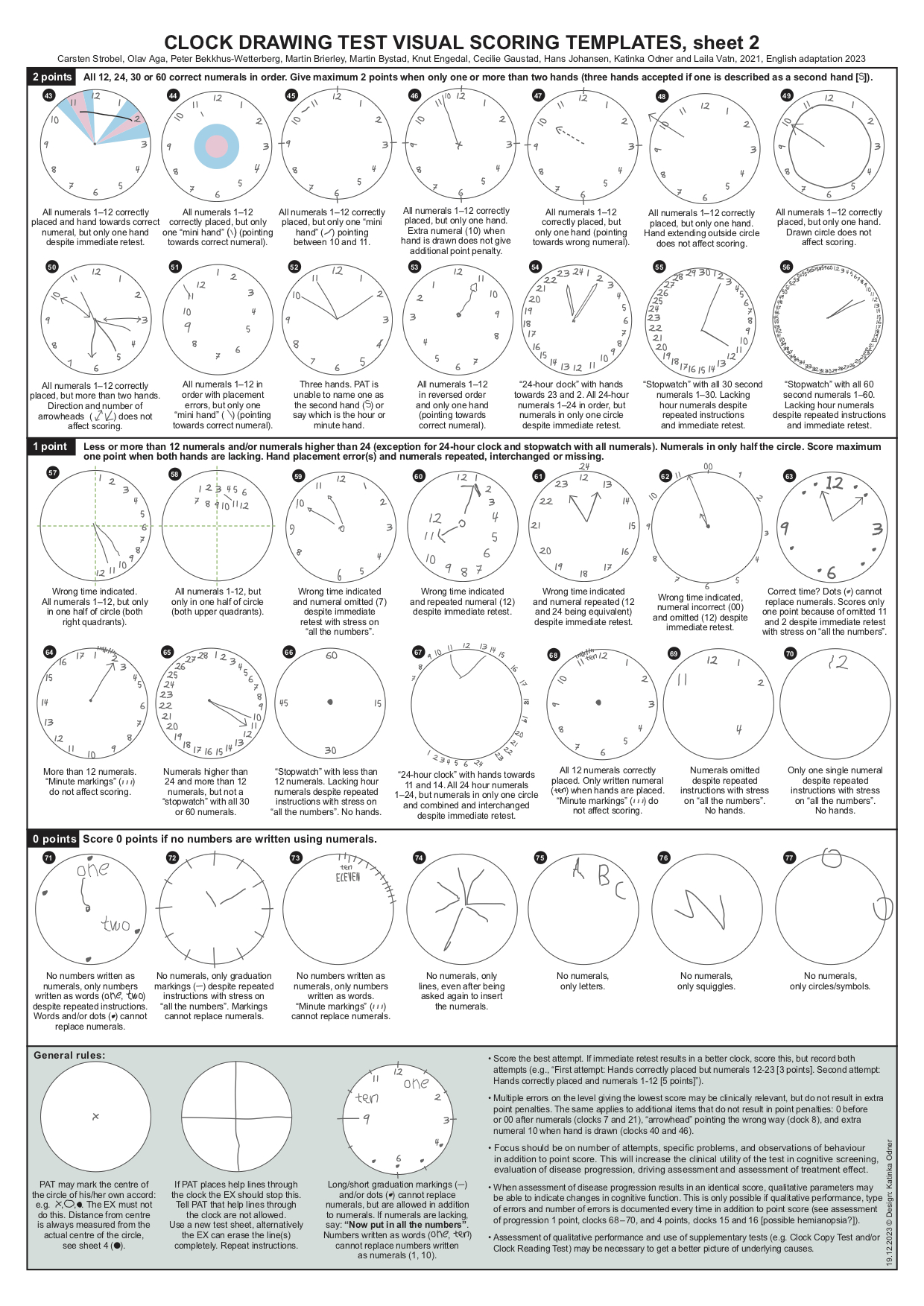


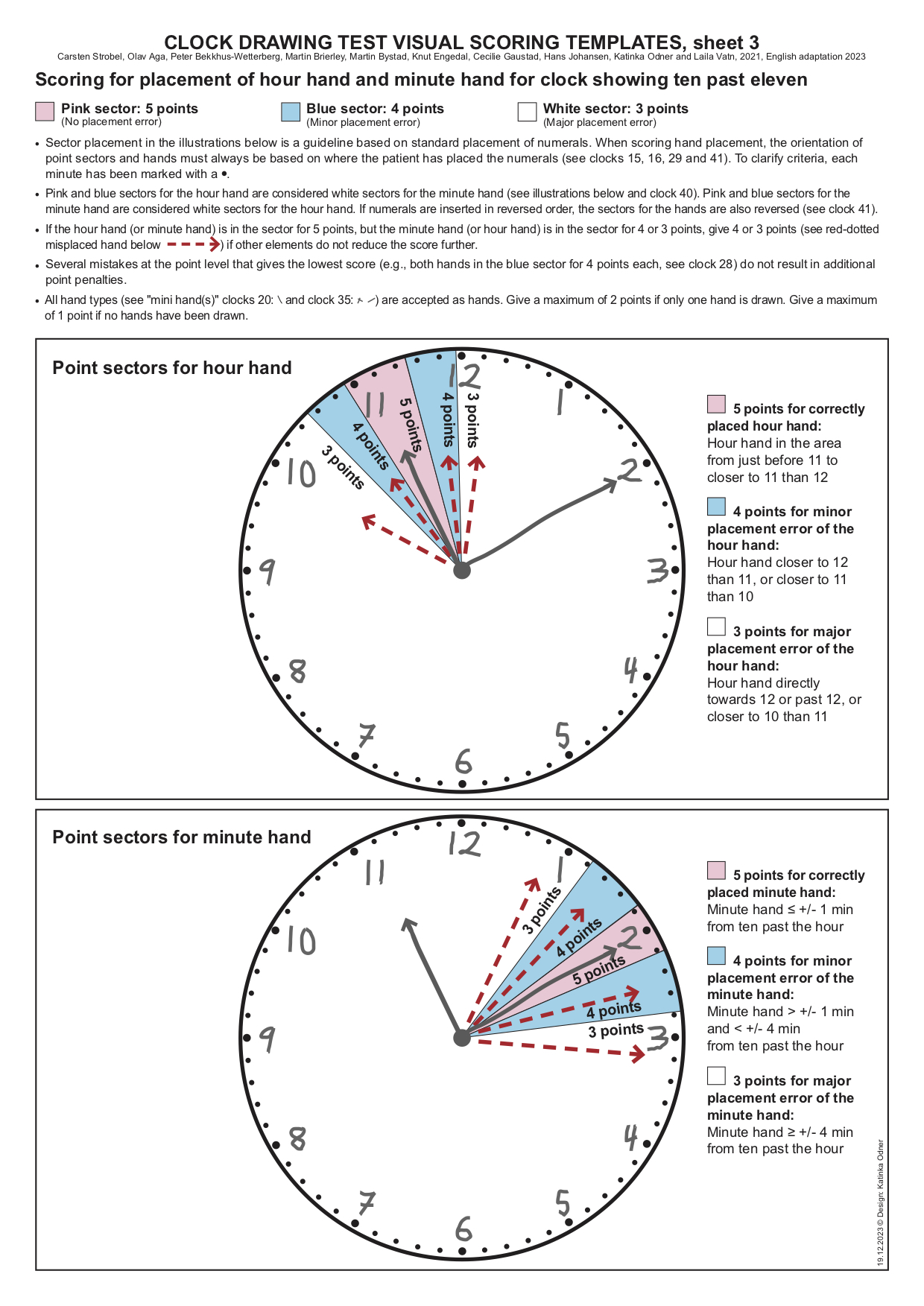

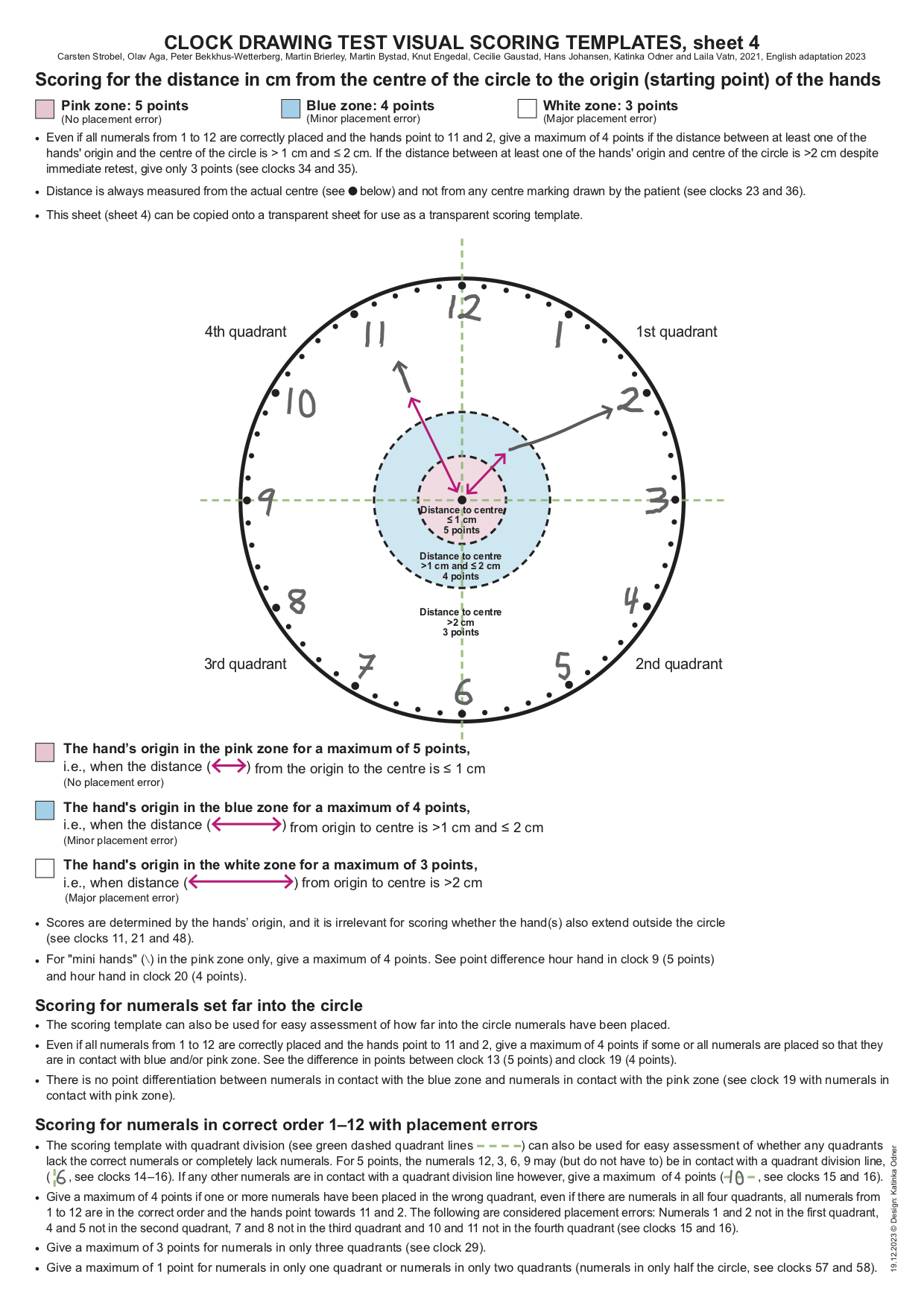


English adaptation by Carsten Strobel, Peter Bekkhus-Wetterberg, Martin Brierly and Katinka Odner.

These visual scoring templates provides examples of different CDT performances and common mistakes yielding different CDT scores. This contributes to increasing both intra- and interrater reliability.

**References**

1. Strobel C, Aga O, Bekkhus-Wetterberg P, Brierley M, Bystad M, Engedal K, et al. Norsk Revidert Klokketest (KT-NR3)

[Norwegian Revised Clock Drawing Test (KT-NR3), English adaptation 2023]: Aldring og Helse, nasjonal kompetansetjeneste; 2021 (cited 21.05.2022) [Available from: <https://www.aldringoghelse.no/wp-content/uploads/2021/09/kt-nr3-testskjema2021-master.pdf>.

2. Strobel C, Aga O, Bekkhus-Wetterberg P, Brierley M, Bystad M, Engedal K, et al. Skåringsillustrasjoner Klokketest [The Clock Drawing Test Visual Scoring Templates, English adaptation 2023]: Aldring og Helse, nasjonal kompetansetjeneste; 2021 (cited 07.10.2023) [Available from: <https://www.aldringoghelse.no/wp-content/uploads/2021/09/skaringsillustrasjoner-klokketest-2021-master.pdf>.
